# The value of plasma pro-enkephalin and adrenomedullin for the prediction of sepsis-associated acute kidney injury in critically ill patients

**DOI:** 10.1186/s13054-020-02887-6

**Published:** 2020-04-21

**Authors:** Ruijin Liu, Xiaoya Zheng, Hongliang Wang, Sicong Wang, Kaijiang Yu, Changsong Wang

**Affiliations:** 1 Department of Critical Care Medicine, Harbin Medical University Cancer Hospital, No. 150 Haping Rd., Nangang District, Harbin, 150081 China; 2 Department of Critical Care Medicine, The Second Affiliated Hospital of Harbin Medical University, Harbin, China; 3 Department of Critical Care Medicine, The First Affiliated Hospital of Harbin Medical University, No. 23 Youzheng Rd., Nangang District, Harbin, 150001 China

Kidney function is commonly affected by sepsis, which is one of the most prominent causes of acute kidney injury (AKI) [1]. Seeking early, reliable biomarkers for the detection of AKI in critically ill patients with sepsis, it is of great importance to conduct successful interventions and reduce adverse outcomes.

Pro-enkephalin is considered as a reliable alternative marker of enkephalin, which is an endogenous opioid peptide and may be associated with decreased renal function [2]. Adrenomedullin, a protein from the calcitonin family, has been reported to increase significantly in patients with sepsis [3]. We prospectively evaluated the predictive value of plasma pro-enkephalin and adrenomedullin (Shuwen Biotech Co. Ltd, China) levels for septic AKI compared to other candidate biomarkers (neutrophil gelatinase-associated lipocalin (NGAL), cystatin-C, kidney injury molecule 1 (KIM-1), and interleukin 18 (IL-18)) (Boster Biological Technology co. Ltd, China).

Forty-two septic patients (age ≥ 18) in the intensive care unit were included in the final analysis. Blood samples were collected from a peripheral vein or central venous within 24 h after the patient was diagnosed with sepsis based on sepsis-3 criteria [4]. According to the 2013 KDIGO standard, patients were divided into one of the cohorts and staged based on the worst serum creatinine and/or the lowest urine output [5].

Among the participants, sixteen patients subsequently suffered from AKI, five of whom were in stage 1, eleven were in stage 3, and no patients were in stage 2. A significant difference of plasma pro-enkephalin concentration exists in the septic AKI group and sepsis group (median 229.2 (93.62–341.2) vs 64.71 (49.23–90.87) pmol/L, *P* < 0.0001). In addition, patients in AKI stage 3 had higher plasma pro-enkephalin (292.94 ± 140.18 vs 125.06 ± 65.82 pg/mL) levels than those in AKI stage 1. The plasma adrenomedullin concentration in the septic AKI group was significantly higher than that in the sepsis group (median 164.69 (118.07–193.52) vs 76.5 (48.66–132.31) pg/mL, *P* = 0.0229). There was no significant difference in plasma NGAL (median 2.37(2.21–2.37) vs 2.25(1.96–3.32) pg/mL, *P* = 0.9631), cystatin-C (mean 28.85 ± 7.25 vs 31.24 ± 14.72 pg/mL, *P* = 0.7353), KIM-1 (median 408.21(0.91–666.5) vs 96.22(0.26–260.12) pg/mL, *P* = 0.1795), and IL-18 (median 136.1(133.36–524.19) vs 184.71(101.84–665.98) pg/mL, *P* = 0.0229) existed in septic patients with and without AKI.

The receiver operating characteristic analysis for the assessment of the diagnostic accuracy of pro-enkephalin and adrenomedullin in the prediction of AKI in septic patients showed significant predictive value for both biomarkers, with area under curve (AUC) of 0.884 (95% CI, 0.738–0.965) and 0.731 (95% CI, 0.560–0.863), respectively (Fig. 1). Pro-enkephalin had a sensitivity of 60.87% and specificity of 100% at the cutoff value of 66.97 pmol/L, while adrenomedullin had a sensitivity and specificity of 75% and 76.92%, respectively, at a cutoff value of 110.44 pg/mL. The combination of the two biomarkers revealed the highest discrimination and had an AUC of 0.890 (95% CI, 0.740–0.969) as well as a sensitivity of 92.31% and a specificity of 69.57%. The pro-enkephalin and adrenomedullin are promising biomarkers for physicians to promptly assess the presence and severity of AKI at an early stage, which will make contribution to start adequate treatment immediately and avoid worse outcome. The limitations of our study include the limited sample size as well as the certain biomarkers, such as NGAL, KIM-1, and IL-18, lacking the detection data of urine specimens which may differ from the diagnostic accuracy of blood specimens.

**Fig. 1:**
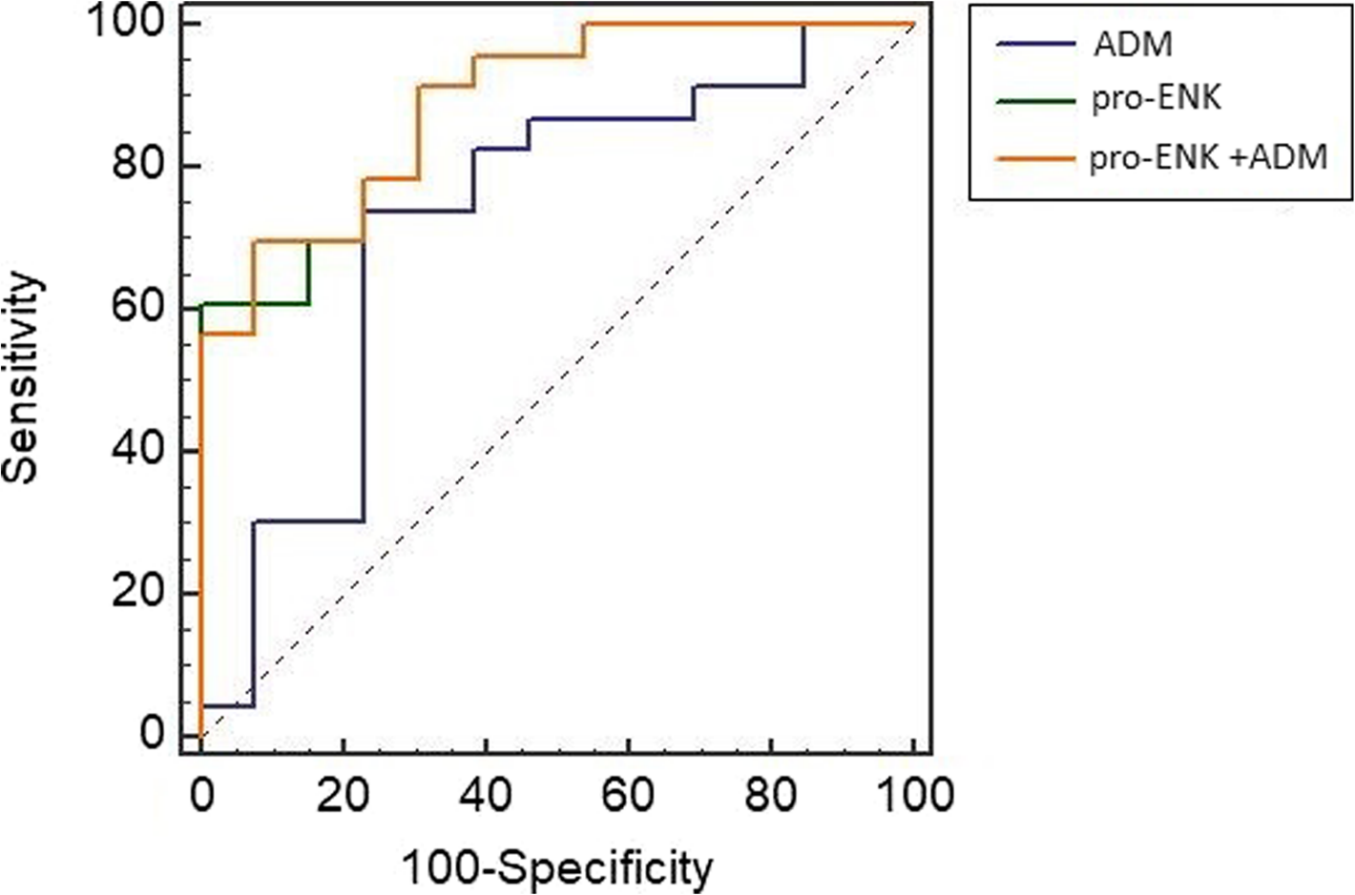
The receiver operating characteristic curve showing the diagnostic power of pro-enkephalin, adrenomedullin, and the combination of pro-enkephalin and adrenomedullin for the estimation of acute kidney injury in septic patients. pro-ENK, pro-enkephalin; ADM, adrenomedullin

## Data Availability

Data are available on request.
